# Flexible Fiber Optic Carbon-Dioxide Laser Assisted Stapedotomy in Otosclerosis

**DOI:** 10.1155/2016/4958074

**Published:** 2016-09-20

**Authors:** Sertac Yetiser

**Affiliations:** Anadolu Medical Center, Department of ORL & HNS, 41400 Kocaeli, Turkey

## Abstract

*Objective*. The aim of this study is to analyze the hearing and vestibular outcome of patients with otosclerosis who have been operated on by fiber optic flexible CO_2_ laser.* Study Design*. A preliminary and retrospective study was conducted in 30 patients with otosclerosis.* Results*. Comparative analysis of average air conduction thresholds (53.41 ± 11.81 dB versus 26.37 ± 11.04 dB) and air-bone gaps (34 ± 9.92 dB versus 12.03 ± 6.02 dB) before and after the surgery were statistically significant (<0.001). Air-bone gap closed within 10 dB or less in 50% of the cases and within 20 dB or less in 90% of the cases. Average bone conduction threshold after the surgery (16.68 ± 12.00 dB) was better than that before the surgery (20.13 ± 8.59). However, no statistically significant difference was found (*p* = 0.213). One patient had tinnitus after surgery. None of the patients had severe sickness or vomiting due to surgery. Eleven patients (36.6%) had very mild nystagmus beating toward the counter-lateral side. All patients were stable at 10 days after surgery.* Conclusion*. The results indicate that fiber optic flexible CO_2_ laser provides the surgeon with a very safe and precise surgical instrumentation even in cases with extensive and obliterative otosclerosis.

## 1. Introduction

Laser stapedotomy was introduced to the otological practice more than 30 years ago [1]. Saccular damage and decrease in cochlear microphonics due to warming of perilymph were major concerns in the early 80s [2–4]. However, the heat in the vestibule has been reported to be harmless if shots with short pulse and low energy are selected and if the shooting is not continuous and the time interval between the shots is longer [5, 6]. One shot of carbon-dioxide laser leads to only 0.3°C increase of heat in the vestibule [7]. Lesinski and Stein reported their first experience about CO_2_ laser surgery in patients with otosclerosis [8]. Thermal effect in CO_2_ is lower and perforation of the oval window is safer due to less tissue penetrance and much absorbance as compared to Argon and KTP [9, 10]. Carbon-dioxide laser has been used for years with a micro manipulator mounted on the microscope under the guidance of a visible light which raised some questions since the surgeon had to use micro mirrors to direct the invisible laser beams to the desired area under the guidance of an aiming beam. Accidental facial paralysis was one of the deleterious effects of the misguided beam [11]. However, introduction of fiber optic CO_2_ laser to the operating field provided significant advance in instrumentation. More accurate shooting was possible by bringing the tip of the applicator over the target area. First experience with fiber-enabled CO_2_ laser has been reported recently [12]. Hand-held laser may provide several practical advantages especially in difficult anatomic conditions like extensive and obliterative otosclerosis.

The aim of this study is to present the preliminary results in auditory and vestibular function in patients with otosclerosis who have been treated with fiber optic flexible carbon-dioxide laser.

## 2. Material and Methods

**Figure 1:**
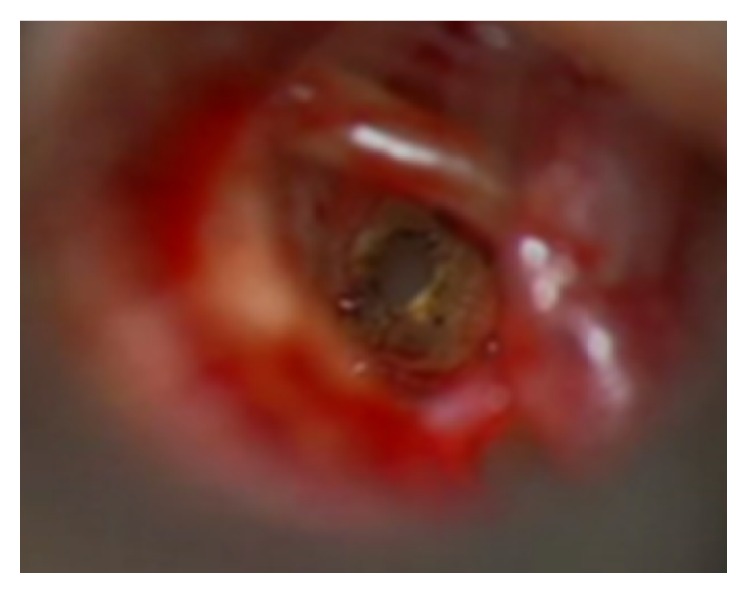
Laser shooting of the footplate.

Thirty patients with otosclerosis who have been operated on between 2009 and 2014 were reviewed. A signed informed consent was obtained from each patient. The procedures were in accordance with the ethical standards of the declaration of Helsinki and of the institutional review board. Diagnosis of otosclerosis was based on the history of progressive conductive hearing loss in the presence of intact tympanic membrane and normal middle ear ventilation. However, footplate fixation was confirmed during surgery. All patients were primary cases. The procedure was performed under general anaesthesia. Classical endomeatal incision was used to elevate the tympanomeatal flap. Flexible fiber optic hand-held carbon-dioxide laser was used (Omni-Guide, Inc., Cambridge, MA, USA) during small fenestra stapedotomy. First, tendon and posterior crus were cut and then penetration of footplate was processed by laser (Figure 1). The power of laser was ranging between 1 and 5 W with pulsed shooting. Shooting time was 0.1 seconds for the tendon and it was ranging between 0.1 and 0.05 seconds for the footplate. None of the shots was exceeding 0.1 seconds. There was an interval of minimum 15 seconds between every shooting. An average of 2-3 shots for the tendon, 4–7 shots for the crus, and footplate were used. Nonoverlapping shooting was used (rosette-fashion) at the footplate. The fenestra was sealed with small pieces of adipose tissue taken from auricular lobule. All patients were subjected to a tinnitus questionnaire, tunning fork test, and nystagmus evaluation a few hours after surgery. Balance was tested the day after surgery and one week later. All patients were discharged on the second postoperative day.

Audiograms taken within two weeks before the surgery were included. Postoperative hearing was tested at one month and every six months after surgery. The most recent hearing test after surgery was included. The follow-up time was maximum 76 and minimum 14 months. Hearing results were described according to the guidelines of the American Committee on Hearing and Equilibrium. Air conduction thresholds at 250, 500, 1000, 2000, 4000, and 6000 Hz and bone conduction thresholds at 500, 1000, 2000, and 4000 Hz were measured. Average air and bone conduction hearing thresholds were calculated as the average of thresholds at 500, 1000, 2000, and 4000 Hz. Air-bone gap at 500, 1000, 2000, and 4000 Hz was calculated as the difference of air and bone conduction thresholds and average air-bone gap was calculated as the average of air-bone gap at those frequencies. Speech reception thresholds and speech discrimination scores were measured before and after surgery. Patients with air-bone gap closure after surgery were grouped as those within 0–10 dB, 11–20 dB, and 21–30 dB. All data obtained before and after surgery were statistically analyzed. “SPSS 15.0 for windows” was used for statistical analysis. Mean values and standard deviations (±) were measured. The one-way ANOVA and Chi-square “goodness of fit” tests were used for comparative analysis of the groups. Statistical significance was set at *p* < 0.05.

## 3. Results

**Figure 2:**
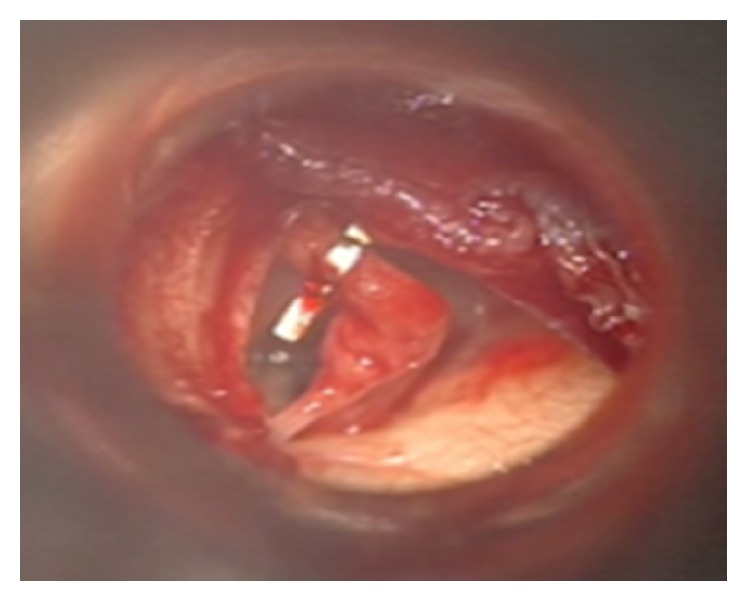
Reverse stapedotomy with preservation of the stapedial tendon.

There were 30 patients (16 males and 14 females) with the average age of 43.1 years (ranging from 21 to 56). The followed-up average time was 25 months (ranging from 14 to 76 months). Surgical intervention was on the left side in 11 and on the right side in 19 patients. 16.6% of patients had tinnitus (number 5) and 6.6% had balance problem before the surgery (number 2). Small fenestra stapedotomy technique was used in all patients and four patients had reverse stapedotomy with preservation of stapedial tendon when there is adequate anatomical access (Figures 1 and 2). Microdrill associated with laser had to be used in 3 patients with extensive obliterative otosclerosis. Maximum number of footplate shot was six. Prosthesis either made of Teflon (number 15) or titanium (number 15) of 0.6 mm diameter was used. The length was ranging between 4 and 4.75 mm. All patients demonstrated hearing improvement and air-bone gap closure after surgery. Comparative analysis of hearing data before and after the surgery is presented in Table 1. Average air conduction threshold and air-bone gap of patients before surgery were 53.41 ± 11.81 dB and 34 ± 9.92 dB, respectively. Twenty patients had pure conductive hearing loss with normal bone conduction before the surgery. Ten patients had 25 dB or worse bone conduction threshold at least at 3 frequencies (mixed type hearing loss). Of those, it was over 30 dB at 3 frequencies in 2 patients (cochlear otosclerosis). Average bone conduction threshold was 20.13 ± 8.59 dB before the surgery.

Air conduction threshold and air-bone gap after the surgery were 26.37 ± 11.04 dB and 12.03 ± 6.02 dB, respectively. Hearing gain for average air conduction and air-bone gap closure after the surgery were statistically significant (<0.001). Average bone conduction threshold after the surgery (16.68 ± 12.00 dB) was better than that before the surgery (20.13 ± 8.59). However, no statistically significant difference was found (*p* = 0.213). One patient with severe obliterative otosclerosis who had required extensive drilling out to achieve fenestration had significant decrease in bone conduction at 4000 Hz from 10 dB to 55 dB. None of the rest of the patients had any drop in bone conduction threshold at any frequency. Air-bone gap was closed within 10 dB or less in 50% of the cases and in 20 dB or less in 90% of cases (Table 2). Speech reception threshold (SRT) improved in all patients after the surgery. The mean speech reception threshold before and after the surgery was 60.68 ± 12.86 dB and 34.34 ± 12.49 dB, respectively, and the difference was statistically significant (<0.001). Average speech discrimination scores (SDS) before and after the surgery were 87.65 ± 19.66 and 88.89 ± 21.88, respectively, and no statistically significant difference was found (*p* = 0.303).

None of the patients had facial paralysis or sensorineural hearing loss or anacusis after the operation. Two patients had additional tinnitus after surgery. Of those, one had no tinnitus ten days after surgery. None of the patients had sickness or vomiting due to surgery. Eleven patients (36.6%) had very mild nystagmus beating toward the contralateral side the day after surgery (Table 3). No spontaneous nystagmus was detected 10 days after operation in any of the patients. All patients were able to walk without any personal help immediately after surgery. However, twelve patients (40%) were not able to do tandem walk the day after surgery. Fifteen patients (50%) had tendency to fall toward the operated side while standing still with closed eyes the day after surgery (Romberg's sign). However, majority of patients demonstrated improved conditions at 10 days after surgery without any medication. Two patients were cured with medication (6.6%). None of the patients had difficulty to do “finger to nose” test the day after surgery (past-pointing).

## 4. Discussion

Stapedotomy with microdrill or pick is sometimes a difficult task to achieve where the anatomy presents challenging conditions such as dehiscent facial nerve, deep oval window, and thick footplate. Besides, it is the surgeon's major concern not to allow extensive trauma to the inner ear. Laser provides nonvibrational penetration of the oval window as compared to microdrilling or manual applications. Functional results in stapedotomy with laser are better than conventional methods. Higher proportion of air-bone gap closure within 10 dB and better bone conduction after the operation has been reported with laser assisted surgery [13–16]. More recently, Fang et al. have conducted a meta-analytic study aiming to compare the hearing outcome of patients with otosclerosis treated with laser and traditional methods. They reported that laser stapedotomy had significantly better hearing outcome [17]. However, thermal effects to the inner ear and associated vestibular symptoms have been subject of discussions [18]. Vestibular function after laser assisted stapedotomy has been less studied. Sakamoto et al. have reported that laser is as safe as conventional technique in terms of duration of vestibular symptoms after the surgery [19]. Almost half of the patients had balance problem the day after surgery in this series, when they are forced to stand still and to walk with closed eyes. But, all improved within a week.

Laser aims ablation of the target tissue by means of heat which is dependent on the thermal and optical properties of the laser itself. This effect is outlined by two major parameters: wavelength and pulse duration. Lower energy inputs will be provided with the presentation of pulsed shootings having cooling intervals [20, 21]. Even in pulsed systems such as Er:YAG laser (Erbium:Yttrium-Aluminum-Oxide), explosive nature of the ablative process raises the risk of acoustic damage [22]. Therefore, success in stapes surgery is mainly dependent on the wavelength property of the laser. Following penetration process of the stapes footplate, CO_2_ has ideal safe conditions for inner ear. Owing to poor absorption in water, there is a risk of damage to deeper-lying structures for KTP and Argon. Comparative analysis of hearing outcome of patients with otosclerosis treated with CO_2_ and other lasers such as KTP-532, Argon, and Er:YAG presents better air-bone gap closure for CO_2_ laser [9, 10, 23, 24]. Air-bone gap closure has been achieved within 20 dB in 90% of cases in this series.

CO_2_ laser can be used as an “one-shot” procedure with increased spot size and power. Jovanovic et al. have optimized the surgical technique with one-shot CO_2_ laser having a footplate perforation of 0.5-0.6 mm to reduce the possible laser-depending inner ear affections. However, adequate diameter could be achieved in 68% of patients [25]. Besides, if a second shot with increased size and power is needed for adequate opening this may increase the risk of inner ear damage [26]. Therefore, fiber-enabled CO_2_ laser with low power and a chance of multiple shooting more accurately is much practical and safer [12]. The fine tip of the laser can be placed over the footplate in a millimetric distance without the need of guided aiming beam. The accuracy problem of the aiming beam from a distance has been one of the disadvantages of CO_2_ laser in the past. Laser mounted on the microscope requires the use of a micromanipulator which is sometimes unable to reach the targeted tissue from a desired angle [12]. Three patients in this series had obliterative otosclerosis which required drilling of the oval window. Obliterative cases present challenging problem and carry the risk for hearing loss and failure. However, it was possible to achieve the oval window fenestration with combined drilling and laser shooting. Conclusively, the results of the study indicate that fiber optic flexible CO_2_ laser provides the surgeon safe and very precise surgical manipulation even in cases with extensive and obliterative otosclerosis.

## Figures and Tables

**Table 1:** Comparative analysis of hearing results of patients with laser stapedotomy before and after surgery.

	Preoperative			Postoperative			*p*
		Min	Max		Min	Max	
AC threshold							
250 Hz	58.96 ± 12.63	35	95	28.79 ± 9.60	10	45	<0.001
500 Hz	56.37 ± 12.31	35	85	26.55 ± 11.26	10	55	<0.001
1000 Hz	52.93 ± 12.78	30	85	25.68 ± 12.79	10	60	<0.001
2000 Hz	49.13 ± 14.58	20	75	28.27 ± 13.84	5	65	<0.001
4000 Hz	50.34 ± 23.67	15	95	37 ± 4120.64	5	90	<0.001
6000 Hz	54.65 ± 29.04	10	110	46.37 ± 21.95	15	95	0.225

Average ACT	53.41 ± 11.81	32	80	26.37 ± 11.04	10	60	<0.001

BC threshold							
500 Hz	17.62 ± 10.08	0	40	13.72 ± 9.19	0	35	0.129
1000 Hz	15.62 ± 9.01	0	30	14.27 ± 10.84	0	40	0.612
2000 Hz	22.06 ± 13.29	5	50	19.48 ± 13.81	0	60	0.473
4000 Hz	21.20 ± 19.42	0	65	21.37 ± 21.10	0	70	1.000

Average BCT	20.13 ± 8.59	5	32	16.68 ± 12.00	0	60	0.213

SRT	60.68 ± 12.86	30	85	34.34 ± 12.49	20	75	<0.001

SDS	87.65 ± 19.66	80	96	88.89 ± 21.88	76	96	0.303

Air-bone gap							
500 Hz	38.44 ± 11.73	15	60	12.96 ± 7.56	0	30	<0.001
1000 Hz	37.93 ± 9.86	25	55	12.20 ± 7.27	0	30	<0.001
2000 Hz	27.41 ± 11.22	5	45	9.13 ± 6.50	0	30	<0.001
4000 Hz	29.48 ± 13.90	5	65	15.93 ± 9.98	0	30	<0.001

Average A-B gap	34 ± 9.92	17	62	12.03 ± 6.02	0	25	<0.001

**Table 2:** Distribution of air-bone gap closure.

ABG closure	Number of patients	Percentage
0–10 dB	15	50
11–20 dB	12	40
21–30 dB	3	10

Total	30	100

**Table 3:** Balance function after surgery in patients with laser stapedotomy.

	Postop day 1		Postop days 7–10	
	Number of patients	Percentage	Number of patients	Percentage
Nystagmus	11	36.6%	None	0%
Tandem walking	12	40%	1	4%
Past-pointing	None	0%	None	0%
Romberg	15	50%	2	6.6%
Additional postop tinnitus	2	8% (25/2)	1	4% (25/1)
